# The Relation Between Brain Amyloid Deposition, Cortical Atrophy, and Plasma Biomarkers in Amnesic Mild Cognitive Impairment and Alzheimer’s Disease

**DOI:** 10.3389/fnagi.2018.00175

**Published:** 2018-06-18

**Authors:** Ling-Yun Fan, Kai-Yuan Tzen, Ya-Fang Chen, Ta-Fu Chen, Ya-Mei Lai, Ruoh-Fang Yen, Ya-Yao Huang, Chyng-Yann Shiue, Shieh-Yueh Yang, Ming-Jang Chiu

**Affiliations:** ^1^Department of Neurology, National Taiwan University Hospital, College of Medicine, National Taiwan University, Taipei, Taiwan; ^2^Institute of Brain and Mind Sciences, National Taiwan University Hospital, College of Medicine, National Taiwan University, Taipei, Taiwan; ^3^PET Center, Department of Nuclear Medicine, National Taiwan University Hospital, College of Medicine, National Taiwan University, Taipei, Taiwan; ^4^Department of Nuclear Medicine, Changhua Christian Hospital, Changhua City, Taiwan; ^5^Molecular Imaging Center, National Taiwan University Hospital, College of Medicine, National Taiwan University, Taipei, Taiwan; ^6^Department of Medical Imaging, National Taiwan University Hospital, College of Medicine, National Taiwan University, Taipei, Taiwan; ^7^PET Center, Tri-Service General Hospital, Taipei, Taiwan; ^8^MagQu Co., Ltd., Xindian District, New Taipei City, Taiwan; ^9^Department of Psychology, National Taiwan University, Taipei, Taiwan; ^10^Graduate Institute of Biomedical Electronics and Bioinformatics, National Taiwan University, Taipei, Taiwan

**Keywords:** plasma Aβ_40_, plasma tau, beta-amyloid, amyloid PET, cortical thickness

## Abstract

**Background:** Neuritic plaques and neurofibrillary tangles are the pathological hallmarks of Alzheimer’s disease (AD), while the role of brain amyloid deposition in the clinical manifestation or brain atrophy remains unresolved. We aimed to explore the relation between brain amyloid deposition, cortical thickness, and plasma biomarkers.

**Methods:** We used ^11^C-Pittsburgh compound B-positron emission tomography to assay brain amyloid deposition, magnetic resonance imaging to estimate cortical thickness, and an immunomagnetic reduction assay to measure plasma biomarkers. We recruited 39 controls, 25 subjects with amnesic mild cognitive impairment (aMCI), and 16 subjects with AD. PiB positivity (PiB+) was defined by the upper limit of the 95% confidence interval of the mean cortical SUVR from six predefined regions (1.0511 in this study).

**Results:** All plasma biomarkers showed significant between-group differences. The plasma Aβ_40_ level was positively correlated with the mean cortical thickness of both the PiB+ and PiB- subjects. The plasma Aβ_40_ level of the subjects who were PiB+ was negatively correlated with brain amyloid deposition. In addition, the plasma tau level was negatively correlated with cortical thickness in both the PiB+ and PiB- subjects. Moreover, cortical thickness was negatively correlated with brain amyloid deposition in the PiB+ subjects. In addition, the cut-off point of plasma tau for differentiating between controls and AD was higher in the PiB- group than in the PiB+ group (37.5 versus 25.6 pg/ml, respectively). Lastly, ApoE4 increased the PiB+ rate in the aMCI and control groups.

**Conclusion:** The contributions of brain amyloid deposition to cortical atrophy are spatially distinct. Plasma Aβ_40_ might be a protective indicator of less brain amyloid deposition and cortical atrophy. It takes more tau pathology to reach the same level of cognitive decline in subjects without brain amyloid deposition, and ApoE4 plays an early role in amyloid pathogenesis.

## Introduction

Although neuritic plaques composed of beta-amyloid (Aβ) fibrils and neurofibrillary tangles (NFTs) consisting of hyperphosphorylated tau protein are the pathological hallmarks of Alzheimer’s disease (AD), the role of brain amyloid deposition in the clinical manifestation or brain atrophy of AD has been debated over decades.

On the one hand (advantages), Aβ deposition has been associated with cognitive decline and neurodegeneration (Frisoni et al., 2009; Villemagne et al., 2013; Sevigny et al., 2016; Iaccarino et al., 2018; Jacobs et al., 2018) or even with emotional (Krell-Roesch et al., 2018) and behavioral symptoms (Marshall et al., 2013; Mori et al., 2014). A recent functional connectivity study showed that brain regions involved in the default mode network (including the precuneus, posterior cingulate, and angular gyrus and medial prefrontal cortex) happen to be among the earliest brain regions of amyloid deposition. Studies also showed that disconnection of the default mode network was related to cognitive dysfunction (Gili et al., 2011) and that functional connectivity and glucose metabolism showed a negative correlation with amyloid burden in nondemented older individuals (Drzezga et al., 2011). On the other hand (disadvantages), Aβ deposition may also be found in some cognitively normal older people (Rowe et al., 2007), even in those already showing cortical thinning (Becker et al., 2011). Functional imaging studies using ^18^F-deoxyglucose-positron emission tomography (PET) showed inconsistent results regarding whether this region showed hypermetabolism or hypometabolism in PiB-positive (PiB+) normal subjects, and a structural study assessing the cortical thickness in these vulnerable brain regions also showed variable results in this group (Oh et al., 2014a). There are still healthy subjects with prominent brain amyloid deposition, either neuritic or diffuse plaques, who remain cognitively unimpaired throughout life (Malek-Ahmadi et al., 2016).

Nevertheless, the development of brain amyloid imaging by PET has advanced dementia research (Villemagne et al., 2013) and clinical trials, especially for AD (Liu et al., 2015; Sevigny et al., 2016). An inverse relation between *in vivo* amyloid deposition measured by ^11^C-Pittsburgh compound B (^11^C-PiB)-PET and cerebral spinal fluid (CSF) Aβ_42_ has long been proposed (Fagan et al., 2006). The relation between CSF Aβ and amyloid deposition has been further explored in subjects with normal cognition and preclinical AD (Racine et al., 2016). On the other hand, blood is an easily accessible biofluid, which can be routinely collected and analyzed repetitively to detect or track the disease. However, studies of the relation between plasma biomarkers and the *in vivo* amyloid deposition measured by ^11^C-PiB-PET still showed mixed results (Kaneko et al., 2014; Tzen et al., 2014; Racine et al., 2016; Fandos et al., 2017; Park et al., 2017). Moving from CSF to blood is a clinical trend of using biomarkers for AD (Blennow, 2017). A reliable blood-based biomarker for AD would be useful in large-scale screening of the general population, diagnosis, and therapeutic decision-making in clinical settings, and longitudinal follow-up of therapeutic effects for disease-modifying therapy in clinical trials (Wang et al., 2017). Our group has developed a superconductivity-based ultrasensitive immunomagnetic reduction (IMR) assay that is reliable for measuring plasma biomarkers of AD and other neurodegenerative diseases (Chiu et al., 2012, 2013; Lee et al., 2016; Yang et al., 2016; Lin et al., 2017).

The epsilon four allele of apolipoprotein (ApoE4) has been identified as an independent risk factor for sporadic AD and a genetic factor that is associated with a lower age at disease onset (Bertram and Tanzi, 2005; Sando et al., 2008). Biomarker studies in subjects with subjective cognitive decline and early mild cognitive impairment (MCI) have revealed increased Aβ deposition and decreased CSF Aβ levels in ApoE4 carriers (Risacher et al., 2013, 2015). In addition, a recent study indicated that there is a positive relationship between plasma Aβ_40_/Aβ_42_ and ^11^C-PiB-PET amyloid deposition (Swaminathan et al., 2014).

Therefore, the aims of the present study are the following: first, to evaluate whether cortical amyloid deposition is related to blood biomarkers and clinical manifestation of dementia; second, to elucidate the relation between amyloid deposition and cortical atrophy in individual brain regions; and third, to explore the effects of ApoE4 carrier status on cortical amyloid deposition in healthy control subjects, subjects with amnesic MCI (aMCI) and subjects with AD.

## Materials and Methods

### Subjects

In this case–control observational study, we included a total of 80 subjects, who were divided into three groups: 39 healthy control subjects, 25 MCI subjects, and 16 AD subjects. Subjects with the diagnoses of aMCI or AD were recruited from the Department of Neurology and Memory Clinic of the National Taiwan University Hospital according to the National Institute on Aging/Alzheimer’s Association Diagnostic Guidelines for MCI due to AD and dementia due to AD (Jack et al., 2011; McKhann et al., 2011; Sperling et al., 2011). Subjects with major systemic diseases, neuropsychiatric disorders, and visual or auditory problems sufficiently severe to interfere with the cognitive function tests were excluded. The demographic data and basic clinical information, including age, gender, education year, Taiwanese Mental Status Examination score, and Clinical Dementia Rating (CDR) score, were collected. A neuropsychological test battery was performed by a board-certified clinical neuropsychologist to help demarcate mild changes in cognition in the subjects with aMCI and in normal daily function. The battery of neuropsychological tests included the Wisconsin Card Sorting Test, Trail Making Test parts A and B, digit span test, logical memory subtests of the Wechsler Memory Scale Version III, Wechsler Adult Intelligence Scale Version III, semantic verbal fluency test, word sequence learning test, visual recognition test, and three-dimensional (3D) block design test as well as a basic language evaluation. The cut-off point for the diagnosis of aMCI was below the fourth percentile of the scaled scores of the age- and education-matched controls of their performance on the neuropsychological tests. The ethics committee of the National Taiwan University Hospital approved the study (201301036 RIND). All subjects and/or their proxies gave their written informed consent before participation in the study. ApoE genotyping was also performed for all participants. All subjects received a ^11^C-PiB-PET scan and underwent a 3D T1-weighted brain magnetic resonance imaging (MRI) scan for the measurements of cortical thickness and subcortical volume.

### PET Image Acquisition and Analysis

Positron emission tomography data were acquired using a PET/CT scanner (Discovery ST4, GE Healthcare, Chicago, IL, United States) with a 2D mode, 47 image planes, and a 15.0-cm axial field of view. After injecting 370–555 MBq of ^11^C-PiB, the PET data were acquired over 30 min (40–70 min postinjection). The emission data were corrected for attenuation, scatter, and radioactive decay, and reconstructed using the ordered subset expectation maximization (OSEM) algorithm with 2 iterations and 15 subsets. A full-width at half-maximum (FWHM) of the 3D Gaussian smoothing kernel (6 mm) was applied to the images in the transverse plane before estimating the realignment parameters. A cranial computer tomography (CT) scan was obtained from each participant before a PET session, so that PET/CT image coregistration could be performed.

The ^11^C-PiB-PET image preprocessing was performed with SPM12 software^1^. First, ^11^C-PiB-PET images were coregistered to the CT brain images and were then further coregistered to the T1-weighted brain MRI template. Second, the T1-coregistered ^11^C-PiB-PET images were normalized to the Montreal Neurological Institute (MNI) space and then smoothed. Third, the amyloid retention in each cortical region was extracted using the MarsBaR toolbox of SPM (Matthew Brett et al., 2002) in the template of the Automated Anatomical Labeling (AAL) atlas. The standard uptake value (SUV) was calculated as the SUV within each cortical region. The mean cerebellar SUV was taken as the average of the cerebellar gray matter (22 regions). Each cortical SUV was subsequently normalized to the mean cerebellar SUV to define the SUV ratio (SUVR) of each cortical region. We calculated the cut-off point for PiB positivity (PiB+) by averaging the SUVRs from the frontal, parietal, lateral temporal, precuneus, anterior, and post-cingulate cortices, and used the upper limit of the 95% confidence interval of the control group as the cut-off point for PiB+, which was 1.0511 in this study (Mormino et al., 2012; Oh et al., 2014b).

### MRI Acquisition and Analysis

High-resolution structural brain MRI scans were acquired using a 1.5-T MRI scanner (EXCITE, General Electric, Milwaukee, WI, United States). A whole-brain T1-weighted 3D spoiled gradient recovery (SPGR) sequence was used (TE = 9.3 ms, TR = 3.9 ms, TI = 600 ms, flip angle = 12°, matrix size = 192 × 192, and FOV = 25 cm), and 170 images of contiguous sagittal slices that were 1.3 mm in thickness were acquired. The analyzed datasets included the T1-weighted structural MRI scans obtained from all of the available subjects (*n* = 80). The MRI image analysis to determine the cortical thickness, gray matter volume, and white matter volume was performed using the FreeSurfer software package (we used the Destrieux cortical atlas with fine parcellation of the cortical areas, we then combined space-related Destrieux cortical areas to approximate an AAL cortical area for comparison with the findings from PET images)^2^.

### Blood Sampling and Assaying of Plasma Biomarkers

#### Blood Sampling and Preprocessing

Approximately 20 ml of whole blood was collected into EDTA-treated tubes. The samples were centrifuged at 2,500 × *g* for 15 min at room temperature within 15 min of the blood draw. The plasma was removed, aliquoted into various volumes, and stored at -80°C. The white blood cell pellets were used for determination of the ApoE genotype. The assays were carried out without knowledge of individual identification or diagnosis. The blood sampling process was arranged to ensure consistency with previous studies using IMR-based assays (Chiu et al., 2012, 2013).

#### Assaying Aβ_42_, Aβ_40_, and Total Tau in Plasma

The reagents used to assay the biomarkers consisted of magnetic nanoparticles coated with dextran and antibodies. All antibody-functionalized magnetic nanoparticles were dispersed in phosphate-buffered saline solution. The magnetic core was Fe_3_O_4_. Depending on the biomarkers, different antibodies were separately conjugated to dextran-coated magnetic nanoparticles. The antibody used for the reagent assaying Aβ_40_ was anti-β amyloid (A3981, Sigma). The antibody used for the reagent assaying Aβ_42_ was anti-β amyloid 37–42 (ab34376, Abcam). The antibody used for the reagent assaying tau was anti-tau (T9450, Sigma). The averaged values of the hydrodynamic diameters of the antibody-functionalized magnetic nanoparticles were approximately 55 nm. The concentration of each kind of reagent was 8 mg Fe/ml.

For IMR measurements, 80 μl of Aβ_1-40_ reagent (MF-AB0-0060, MagQu) was mixed with 40 μl of sample, 60 μl of Aβ_1-42_ reagent (MF-AB2-0060, MagQu) was mixed with 60 μl of sample, and 80 μl of tau reagent (MF-TAU-0060, MagQu) was mixed with 40 μl of sample for assaying Aβ_1-40_, Aβ_1-42_, and tau, respectively. Duplicated measurements were conducted for each biomarker per sample. The measurements were performed with an IMR analyzer (XacPro-S, MagQu). For further technological details, please refer to our previous work (Yang et al., 2011, 2017; Chiu et al., 2012, 2013).

### Statistical Analysis

Parametric variables, including age, education years, TMSE scores, and neuroimaging measures, were analyzed and examined for intergroup differences using analysis of variance (ANOVA) with *post hoc* test using Bonferroni method. Nonparametric data, such as gender, ApoE4 carrier status, and brain amyloid positivity, were compared using the Pearson Chi-square test for intergroup differences. Multivariate analysis of covariance (MANCOVA), using age as the covariate, was applied to examine all of the cortical regions of interest (ROIs) for significant between-group differences in cortical amyloid deposition and cortical thickness with a false-discovery rate (FDR) correction for type I error (Benjamini–Hochberg procedure) for multiple comparisons.

Lastly, the Spearman correlation analysis was used to clarify the correlation between plasma levels of biomarkers, cortical thickness, and amyloid deposition in subjects with high amyloid deposition, designated the PiB+ group, and in those with low amyloid deposition, designated the PiB– group, respectively. We further examined the contribution of plasma biomarkers and amyloid deposition to cortical thickness by using stepwise linear regression. All statistical analyses were performed by using SPSS software version 22.0 (IBM, Armonk, NY, United States).

## Results

### Between-Group Differences in Plasma Biomarkers, Brain Amyloid Deposition, and Cortical Thickness

**FIGURE 1:**
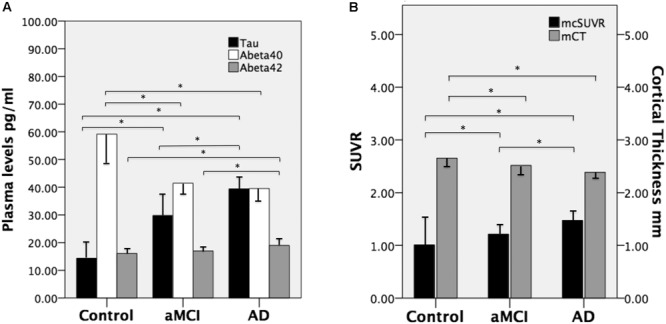
**(A)** Mean plasma levels of tau, Aβ_40_, and Aβ_42_ in the three groups and the between-goups and the between-group differences (^∗^ indicates *P* ≤ 0.001). **(B)** The mean cortical thickness in millimeters (mCT) and mean cortical SUVR (mcSUVR) in the three groups and the between-group differences (^∗^ indicates *P* < 0.001). aMCI, amnesic mild cognitive impairment and AD, Alzheimer’s disease. T bars (inversed T bars) stand for 1 standard deviation of each biomarker of each group.

There were no significant between-group differences in age, education years, and gender distribution (**Table 1**). The percentage of ApoE4 carrier status was highest in the AD group, followed by the aMCI group and the control group (Pearson Chi-square 6.348, *P* = 0.042). The plasma biomarkers showed significant between-group differences in levels of total tau, Aβ_40_, and Aβ_42_: *post hoc* test using Bonferroni method further showed that plasma tau levels were significantly different between the control group and both the aMCI and AD groups (both *P* < 0.001), and the plasma tau levels between the aMCI group and the AD group also showed a significant difference (*P* = 0.001) (control < aMCI < AD); plasma levels of Aβ_40_ were significantly different between the control group and both the aMCI and AD groups (both *P* < 0.001) but not between the aMCI group and the AD group (control > aMCI and control > AD); plasma Aβ_42_ levels were significantly different between the AD group and both the control and aMCI groups (both *P* < 0.001) but not between the aMCI group and the control group (**Table 2** and **Figure 1A**) (control < AD and aMCI < AD).

**Table 1:** Demographic and clinical information of the three groups.

Groups (number)	Control (39)	aMCI (25)	AD (16)	^a^*F*^b^*Chi*	*P*
Age (years old)	63.0 ± 8.5	68.0 ± 9.8	67.6 ± 12.2	2.474^a^	0.091
Education (years)	12.2 ± 4.8	11.6 ± 5.0	11.7 ± 3.8	0.138^a^	0.871
TMSE	28.8 ± 1.1	26.6 ± 3.1	16.0 ± 6.0	27.814^a^	<0.001
Gender (F/M)	23/16	11/14	7/9	1.818^b^	0.403
CDR (0/0.5/1)	39/0/0	0/21/0	0/9/7	105.735^b^	<0.001
ApoE**𝜀**4 carriers (%)	9/39 (23.1)	11/25 (44)	9/16 (56.2)	6.348^b^	0.042

*^*a*^Analyses of variance. ^*b*^Pearson’s Chi-square test. ApoE**𝜀**4 carrier (% of the group). aMCI, amnesic mild cognitive impairment; AD, Alzheimer’s disease; TMSE, Taiwanese Mental State Examination; CDR, Clinical Dementia Rating.*

**Table 2:** Biomarkers of the three groups.

Groups (number)	Control (39)	aMCI (25)	AD (16)	^a^*F*^b^*Chi*	*P*
PiB+/PiB-	11/28	17/8	15/1	22.577^b^	<0.001
PiB+ of E4- (%)	6 (20)	7 (50)	6 (100)	16.18^b^	<0.001
PiB+ of E4+ (%)	5 (55.6)	10 (90.9)	9 (88.9)	4.5^b^	=0.105
^mc^SUVR	1.01 ± 0.66	1.21 ± 0.23	1.47 ± 0.27	38.199^a^	<0.001
^m^CT (mm)	2.65 ± 0.19	2.52 ± 0.18	2.39 ± 0.14	13.81^a^	<0.001
Plasma Aβ_40_ (pg/ml)	59.2 ± 11.1	41.5 ± 3.9	39.5 ± 5.8	47.634^a^	<0.001
Plasma Aβ_42_ (pg/ml)	16.1 ± 1.8	17.0 ± 2.0	19.0 ± 2.7	11.192^a^	<0.001
Plasma Tau (pg/ml)	14.3 ± 6.4	29.7 ± 8.7	39.4 ± 5.8	61.535^a^	<0.001

*^*a*^Analyses of variance. ^b^Pearson’s Chi-square test. aMCI, amnesic mild cognitive impairment; AD, Alzheimer’s disease; PiBC+/PiB-, ratio between subjects with PiB-PET positivity and negativity; TMSE, Taiwanese Mental State Examination; ^m^CT, mean thickness of all cortical areas in millimeters; ^mc^SUVR, mean SUVR of all cortical regions; E4+, ApoE4 carrier; E4-, non-ApoE4 carrier.*

We calculated the mean SUVR of all cortical regions to obtain the mean cortical SUVR (^mc^SUVR) for each subject. There was a significant between-group difference for ^mc^SUVR (*P* < 0.001). The *post hoc* analyses showed significant differences between all three groups (all *P* < 0.001; **Table 2**) (control < aMCI < AD). In a similar vein, we calculated the mean cortical thickness of all cortical regions (^m^CT) for each subject. There was a significant between-group difference for ^m^CT (*P* < 0.001); *post hoc* analyses showed significant differences between the control group and both the aMCI (*P* = 0.01) and AD groups (*P* < 0.001) but not between the aMCI group and the AD group (*P* = 0.074; **Table 2** and **Figure 1B**) (control > aMCI and control > AD).

### Relation Between Plasma Biomarkers, Cortical Thickness, and Brain Amyloid Deposition

First, we examined the relation between plasma biomarkers and ^m^CT in both the PiB+ group (*n* = 42) and PiB- group (*n* = 38). Plasma Aβ_40_ levels were positively correlated with ^m^CT in both the PiB+ (*rho* = 0.457, *P* = 0.002) and PiB- groups (*rho* = 0.351, *P* = 0.031). Plasma tau levels were negatively correlated with ^m^CT in both the PiB+ group (*rho*= -0.59, *P* = 0.02) and PiB- group (*rho*= -0.322, *P* = 0.048). Plasma Aβ_42_ levels were negatively correlated with ^m^CT in the PiB- group (*rho*= -0.338, *P* = 0.038) but not in the PiB+ group (*rho*= -0.051, *P* = 0.750).

Second, we examined the relation between plasma biomarkers and ^mc^SUVR in both the PiB+ group and PiB- group. Plasma Aβ_40_ levels were negatively correlated with ^mc^SUVR in the PiB+ group (*rho* = -0.585, *P* < 0.001) but not in the PiB– group (*rho* = -0.203, *P* = 0.221). Plasma tau levels showed a significant positive correlation with ^mc^SUVR in the PiB+ group (*rho*= 0.422, *P* = 0.003) but not in the PiB- group (*rho* = 0. 126, *P* = 0.451). Plasma Aβ_42_ levels were not significantly correlated with ^mc^SUVR in both the PiB– group (rho = -0.295, *P* = 0.072) and PiB+ group (*rho*= -0.281, *P* = 0.072).

In summary, there was no significant correlation between plasma biomarkers and ^mc^SUVR in the PiB(-) group.

Then, we examined the relation between ^m^CT and ^mc^SUVR. ^m^CT was negatively correlated with ^mc^SUVR (*rho* = -0.506, *P* = 0.001) in the PiB+ group but not in the PiB- group (*rho* = 0.04, *P* = 0.81). We also found a negative correlation between MMSE scores and plasma tau levels in the PiB+ group (*rho* = -0.624, *P* < 0.001) but not in the PiB- group (*rho* = -0.188, *P* = 0.259). Age was not significantly correlated with plasma biomarkers, ^m^CT, or ^mc^SUVR in PiB+ group or the PiB- group.

We further examined the contribution of plasma biomarkers and ^mc^SUVR (independent variables) to ^m^CT (dependent variable) by stepwise linear regression analysis. In Supplementary Figure 1, univariate linear regression of the plasma biomarkers and ^mc^SUVR is shown, demonstrating a linear relation between plasma biomarkers, amyloid deposition, and cortical atrophy. Age and ApoE4 loading were also included as covariates in the stepwise linear regression analysis. The results showed that ^mc^SUVR contributed the most to ^m^CT accounting for about 20% of the factor (*R square change* 0.210, *F* 20.678, *P* < 0.001, β -0.305 Model II) followed by plasma Aβ_40_ level, which explained additional 6.5% (*R square change* 0.065, *F* 6.895, *P* = 0.010, β 0.297 Model II). Finally, we examined the contribution of ^mc^SUVR (independent variables) to plasma tau levels (dependent variable) by stepwise linear regression analysis with age, ApoE4 loading, and ^m^CT included as covariates. We found that ^mc^SUVR was the only contributing factor explaining more than 25% (*R square change* 0.257, *F* 26.978, *P* < 0.001, β 0.507 Model I) for plasma tau levels.

### Receiver Operating Curve Analyses of Plasma Biomarkers and Amyloid Deposition

In the PiB- group, the cut-off points of plasma biomarkers for differentiating between controls and subjects with AD are much higher (tau 37.54 pg/ml and Aβ_42_ 21.92 pg/ml) than those of their PiB+ counterparts (tau 25.57 pg/ml and Aβ_42_ 16.81 pg/ml) (**Table 3**). On the other hand, the ^mc^SUVR cut-off point of the PiB+ subjects (1.2167) is higher than that of their PiB- counterparts (1.0143).

**Table 3:** Results of the ROC analyses of plasma biomarkers and amyloid deposition.

Group (number)	Biomarkers	AUC	Cut-off point	Sensitivity (%)	Specificity (%)
All (80)	Tau	0.974	26.56 pg/ml	93.8	100
	Aβ_42_	0.829	16.99 pg/ml	87.5	84.6
	^mc^SUVR	0.974	1.0942	93.8	94.9
PiB- (38)	Tau	1.000	37.54 pg/ml	100	100
	Aβ_42_	1.000	21.92 pg/ml	100	100
	^mc^SUVR	0.893	1.0143	100	89.3
PiB+ (42)	Tau	0.976	25.57 pg/ml	93.3	100
	Aβ_42_	0.776	16.81 pg/ml	86.7	81.8
	^mc^SUVR	0.988	1.2167	93.3	100

*ROC, receiver operating curve analyses differentiating between controls and patients with Alzheimer’s disease; AUC, area under the curve of ROC analysis; PiB+, subjects categorized with positive amyloid deposition; ^*mc*^ SUVR, mean SUVR of all cortical regions.*

Aβ_40_ have low AUC (0.058) thus a useful cut-off point is not available. From the above receiver operating curve (ROC) analyses we may conclude that plasma tau levels and Aβ_42_ levels together with ^mc^SUVR are useful biomarkers for differentiating controls and subjects with AD.

### Region-Specific Between-Group Differences in Cortical Thickness and Amyloid Deposition and Their Relation

There were significant region-specific between-group differences in cortical thickness in most regions (all *P* < 0.009, FDR adjustment) except in the bilateral orbital frontal gyri, fusiform gyri, anterior cingulate, and right inferior frontal gyrus and lingual gyrus (**Table 3**). On the other hand, there were significant region-specific between-group differences in amyloid deposition in most regions (all *P* < 0.009, FDR adjustment) except in the bilateral temporal poles and inferior temporal gyri (**Table 3**).

The relationship between amyloid deposition and cortical thickness (designated atrophy due to a negative correlation) could be categorized into two groups according to their levels of correlation. Group I was the group with relatively high (compared to the rest of the analyzed regions) levels of correlation (H in **Table 4**) and a designated significance level of *P* < 0.005 on either side of the cortical region, with the Spearman *rho* ranging from -0.317 to -0.514 (Supplementary Table 1). The cortical regions in group I were heteromodal isocortices, which also had significant between-group differences in cortical thickness and amyloid deposition. Group II consisted of either low levels of correlation, with the Spearman *rho* ranging from -0.070 to -0.215 (*P* > 0.05) on both sides, or moderate levels of correlation, with the Spearman *rho* ranging from -0.221 to -0.314 (*P* < 0.05 but *P* ≥ 0.005 on either side; Supplementary Table 1). Group II was further classified phylogenetically into three subgroups, IIa limbic (allocortex), IIb paralimbic (mesocortex or periallocortex), and IIIc proisocortex (unimodal neocortex that abuts the insula and parahippocampus) (**Table 4**). In group-IIa, both the hippocampus and amygdala had significant brain atrophy and amyloid deposition, while the correlation levels between atrophy and deposition were low for amygdala and moderate for hippocampus. In group-IIb, except the temporal poles, all regions had significant between-group differences in amyloid deposition. On the other hand, the brain atrophy was variable, and there was no significant atrophy in the frontal orbital gyri and anterior cingulate gyri. In group-IIc, all but the inferior temporal gyri had significant between-group differences in amyloid deposition. The fusiform gyri had no significant between-group difference in cortical thickness.

**Table 4:** Grouping of regions by significance of amyloid deposition, cortical atrophy, and correlation.

Group	Atrophy	Amyloid	Corr.	
I	H	H^∗^	H	Frontal sup, mid; angular, supramarginal, precuneus, parietal sup, temporal mid, inf^∗^
II	Variable^#^	H^∗^	L or M	a. Hippocampus, amygdala
				b. Parahippocampus, frontal orb^#^; cingulate ant^#^, mid, post; temporal pole^∗^
				c. Temporal sup, and frontal in., lingual, fusiform^#^

*Atrophy: significant level of between-group differences in cortical thickness, designated cortical, or brain atrophy of a specific region. Amyloid: significant level of between-group differences in amyloid deposition, designated amyloid load of a specific region. Ant, anterior; mid, middle; post, posterior; sup, superior; inf, inferior. Group I are heteromodal isocortex; Group IIa are limbic (allocortex); IIb are paralimbic (mesocortex or periallocortex); IIc are unimodal proisocortex abutting insula and parahippocampus. ^∗^No significant between-group difference in amyloid deposition. ^#^No significant between-group difference in cortical thickness. H was designated a significance level of *P* < 0.005 on either side of the cortical region, with the Spearman rho ranging from -0.317 to -0.514; for hippocampus and amygdala volumes were used for between group comparison.*

### ApoE4 Increased Cortical Amyloid Deposition in the Controls and the aMCI Group

The percentage of PiB+ was higher in both the aMCI and AD groups than in the control group; ApoE4 carrier status increased the rate of PiB+, especially in the aMCI group and the control group (**Table 2**).

## Discussion

### Amyloid Deposition Accelerates Cortical Atrophy, but Plasma Aβ_40_ Might Be a Protective Indicator

We demonstrated in this study that cortical amyloid deposition, in terms of ^mc^SUVR, explains more than 20% of the cortical thickness. In the same vein, ^mc^SUVR explains more than 25% of the plasma tau levels, which are surrogate biomarkers of neuroaxonal injury due to neurodegeneration. Cortical thickness was negatively correlated with amyloid deposition, plasma tau was negatively correlated with cognitive decline but positively with amyloid deposition only in the PiB+ group but not in the PiB- group. These findings support the hypothesis that Aβ may increase the accumulation of tau aggregates and, in particular, accelerate the spread of tau into the isocortex with the consequence of neuronal loss, cortical atrophy (R. Sperling et al., 2014) and cognitive decline. Numerically, the lack of associations between plasma biomarkers and cerebral amyloid deposition in the PiB- group could be best explained by the floor effect of the ^mc^SUVR in this group.

Then, we found that in the PiB+ group, plasma Aβ_40_ was negatively correlated with amyloid deposition (*r* = -0.510, *P* = 0.001), although amyloid plaques in Alzheimer’s pathology mainly consist of Aβ_42_ despite the fact that Aβ_40_ is several fold more abundant in the brain as well as the plasma (Hardy and Selkoe, 2002; Chiu et al., 2012; Tzen et al., 2014; Economou et al., 2016). Some studies showed that although Aβ_40_ fibrils may participate in the formation of amyloid plaques, the aggregation of Aβ_40_ fibrils is too slow to form insoluble aggregates to account for amyloid deposition in AD (McGowan et al., 2005). In a transgenic mouse study, intraneuronal Aβ_42_ but not Aβ_40_ was found to lead to cellular Aβ lesions (Abramowski et al., 2012). This might suggest that there is dissociated neuronal toxicity between these two major alloforms of Aβ, i.e., Aβ_42_ versus Aβ_40_ (Sengupta et al., 2016). In addition, plasma Aβ_40_ levels also showed a positive correlation with total cortical thickness in both the PiB+ group (*rho* = 0.510, *P* = 0.001) and PiB- group (*rho* = 0.402, *P* = 0.007). This might imply not only that Aβ_40_ is less toxic in the brain but also that the plasma Aβ_40_ level may be a protective indicator in terms of less cortical atrophy and less amyloid pathology. The effect of age on cortical thickness was significant only in the control group (*rho* = -0.356, *P* = 0.026), which is in agreement with previous studies (Thambisetty et al., 2010; Hurtz et al., 2014). Cortical atrophy may be attributed more to disease severity than to age within the prodromal or early dementia stage of AD.

### Elevated Cut-off Point of Plasma Tau Level in PiB- Group

In *PiB- group*, the cut-off point of the plasma tau level (37.54 pg/ml) is much higher than in their amyloid positive counterparts (25.57 pg/ml). This might suggest that without the participation of amyloid plaques in the brain, total plasma tau levels, a surrogate biomarker of neuronal loss, would be elevated to reach the same level of cognitive decline (Rosen et al., 2013; Tatebe et al., 2017).

### Amyloid Deposition Precipitates Cortical Atrophy in AD-Signature Isocortex

In this study, we found significant between-group differences in cortical thickness (cortical atrophy) in the bilateral hippocampus; parahippocampus; amygdala; middle and posterior cingulate, angular, and supramarginal gyrus; precuneus; and bilateral temporal, superior, and middle frontal cortices, which is in accordance with AD-signature areas (Dickerson et al., 2009; Abu-Raya et al., 2016). Furthermore, our results revealed cortical thinning over multimodal association cortical regions, such as the angular gyrus, supramarginal gyrus, precuneus, bilateral temporal cortices, and superior and middle frontal cortices, which are phylogenetically heteromodal isocortices (**Table 4**). In contrast, the hippocampus, amygdala, and cingulate cortices are allocortex (limbic) or periallocortex (paralimbic) phylogenetically, and the atrophy in these regions is much less dependent on the existence of amyloid protein (**Table 4**).

According to previous studies, some AD-signature regions with cortical thinning primarily contribute by accumulating NFTs, whereas in other regions, both amyloid plaques and NFTs account for cortical atrophy (Arnold et al., 1991; Braak and Braak, 1991). For instance, NFT accumulation in the medial temporal lobe and particular perirhinal and entorhinal cortex could cause regional atrophy, even in the absence of Aβ deposition (Braak et al., 2011). These differential vulnerabilities may have resulted from different cellular stress susceptibilities to toxic proteinopathy (Posimo et al., 2015). Recent PET studies supported the idea that tau deposition in isocortical regions might require the presence of Aβ but not in the allocortical region (Scholl et al., 2016; Sepulcre et al., 2016).

### ApoE4 and Cortical Amyloid Deposition

Our results revealed that amyloid depositions were affected by ApoE4 carrier status, which is compatible with previous findings (Reiman et al., 2009), supporting the concept that ApoE4 plays an early role in the pathogenesis of AD. ApoE4 manifests its influences by increasing the prevalence of PiB+ in both the control and aMCI groups. The ApoE4 effect was not significant in the AD group, probably due to the already widespread amyloid plaques at this clinical stage. Several potential mechanisms linking ApoE4 and the Aβ process have been proposed, for example, ApoE would affect Aβ clearance and aggregation (Bell et al., 2007; Deane et al., 2008; Cerf et al., 2011; Kanekiyo et al., 2014), protease-dependent degradation of Aβ is enhanced by various ApoE isoforms (Saido and Leissring, 2012), and cellular uptake of Aβ is influenced by ApoE (Cudaback et al., 2011). Thus, we may conclude that ApoE4 plays an early role in the pathogenesis of amyloid deposition in AD.

### Study Limitations

There are some limitations to this study. First, this was a retrospective, case–control observational study. We did not observe longitudinally whether the subjects who showed positive amyloid deposition experienced more rapid cortical atrophy than did those without amyloid deposition. Second, tau imaging was lacking, and such imaging could have directly revealed the role of tau in cortical thickness reduction. Lastly, the SUVRs were normalized to an MNI 152 space by SPM while the morphometric measures were normalized to MNI 305 by FreeSurfer. These two coordinates may not match each other; we could only use the combination of Destrieux atlas to approximate the AAL areas. This is an important methodological constraint, which should be beared in mind when we are interpretating or generalizing the image data from this report.

In the future, a large-scale longitudinal study with subjects with different disease severity is warranted.

## Conclusion

Our study demonstrated that the contributions of amyloid deposition to AD pathology and cortical thickness are spatially distinct. Plasma Aβ_40_ might be a protective indicator in terms of less amyloid pathology and cortical atrophy. It takes more tau pathology to reach the same level of cognitive decline in subjects without much amyloid deposition. Finally, ApoE4 plays an early role in amyloid pathogenesis.

## Ethics Statement

This study was carried out in accordance with the recommendations and guidelines by the ethics committee of our university hospital with written informed consent from all subjects. All subjects gave written informed consent in accordance with the Declaration of Helsinki. The protocol was approved by the Ethics Committee of our University Hospital.

## Author Contributions

L-YF drafted the manuscript, processed the image data, and performed the statistical analyses. K-YT performed the acquisition and preprocessing of the PET data and revised the manuscript. Y-FC performed the acquisition and preprocessing of the MRI data. T-FC recruited the clinical patients and processed the clinical information. Y-ML performed the neuropsychological tests and preprocessing of the data. R-FY performed the acquisition and preprocessing of the PET data. Y-YH and C-YS monitored the quality control of ^11^C-PiB production and provided advice for the amyloid PET scans. S-YY supervised the measurement of plasma biomarkers. M-JC was responsible for the research concept initiation, study design, data quality, manuscript revisions, and final approval.

## Conflict of Interest Statement

S-YY is an employee of MagQu Co., Ltd., Xindian District, New Taipei City, Taiwan. The remaining authors declare that the research was conducted in the absence of any commercial or financial relationships that could be construed as a potential conflict of interest.

## Footnotes

1 http://www.fil.ion.ucl.ac.uk/spm/

2 http://surfer.nmr.mgh.harvard.edu/
