# Supplementary material for: The Relation Between Brain Amyloid Deposition, Cortical Atrophy, and Plasma Biomarkers in Amnesic Mild Cognitive Impairment and Alzheimer’s Disease
Source: Front Aging Neurosci. 2018 Jun 18;10:175. doi: 10.3389/fnagi.2018.00175 (PMC6015901; doi:10.3389/fnagi.2018.00175)
Supplement: Supplementary file 1 [file Data_Sheet_1.pdf]

Supplementary Table 1. Comparison of between-group differences in regional cortical thickness and amyloid deposition and their correlation

	Cortical thickness (*F*)		Amyloid deposition (*F*)		Correlation (*rho*)	
Brain regions	Left	Right	Left	Right	Left	Right
Isocortex (true isocortex and proisocortex)						
Frontal sup	6.9*	5.808*	33.439*	39.482*	-0.334[c]	-0.334[c]
Frontal mid	10.948*	8.51*	32.516*	36.859*	-0.287[a]	-0.352[c]
Frontal inf	6.645*	4.803	20.467*	15.681*	-0.210	-0.215
Parietal sup	5.12*	10.654*	37.649*	31.782*	-0.267[a]	-0.317[c]
Angular	10.196*	15.824*	26.252*	27.784*	-0.448[d]	-0.368[c]
Supramarginal	6.846*	8.642*	29.903*	25.684*	-0.362[c]	-0.319[c]
Precuneus	11.261*	8.853*	38.233*	39.891*	-0.368[c]	-0.417[d]
Temporal sup	10.352*	11.293*	31.515*	29.186*	-0.301[b]	-0.265[a]
Temporal mid	11.521*	15.253*	25.775*	18.413*	-0.351[c]	0.330[c]
Temporal inf	10.581*	10.726*	4.710	3.586	-0.514[d]	-0.333[c]
Fusiform	3.809	1.235	15.313*	19.223*	-0.196	-0.070
Lingual	7.078*	4.777	19.734*	19.703*	-0.203	-0.081
Paralimbic (mesocortex, periallocortex)						
Cingular ant	3.89	2.965	33.923*	48.778*	-0.122	-0.150
Cingular mid	6.379*	5.599*	24.66*	24.025*	-0.013	-0.091
Cingular post	11.148*	9.774*	20.467*	15.881*	-0.221[a]	-0.180
Temporal pole	2.013	9.7*	4.879	0.711	-0.156	-0.224[a]
Frontal orb	0.391	1.374	10.459*	10.156*	-0.285[a]	-0.306[b]
Parahippocampus	12.845*	8.957*	13.457*	13.786*	-0.294[b]	-0.314[b]
Limbic (allocortex)						
Hippocampus	24.383*	26.441*	17.088*	11.345*	-0.313[b]	-0.222[a]
Amygdala	6.843*	9.439*	7.431*	8.893*	0.110	-0.129

F values of cortical thickness and amyloid deposition from multi analysis of variance; *rho* for Spearman correlation between regional cortical thickness (or volume) and amyloid deposition of the corresponding area; ant: anterior; mid: middle; post: posterior; sup: superior; inf: inferior; *: Significant between-group differences $P \leq 0.009$ with FDR (false discovery rate) correction for multiple comparisons; [a] $P < 0.05$; [b] $P < 0.01$; [c] $P < 0.005$; [d] $P < 0.001$; for hippocampus and amygdala volumes were used for between group comparison.

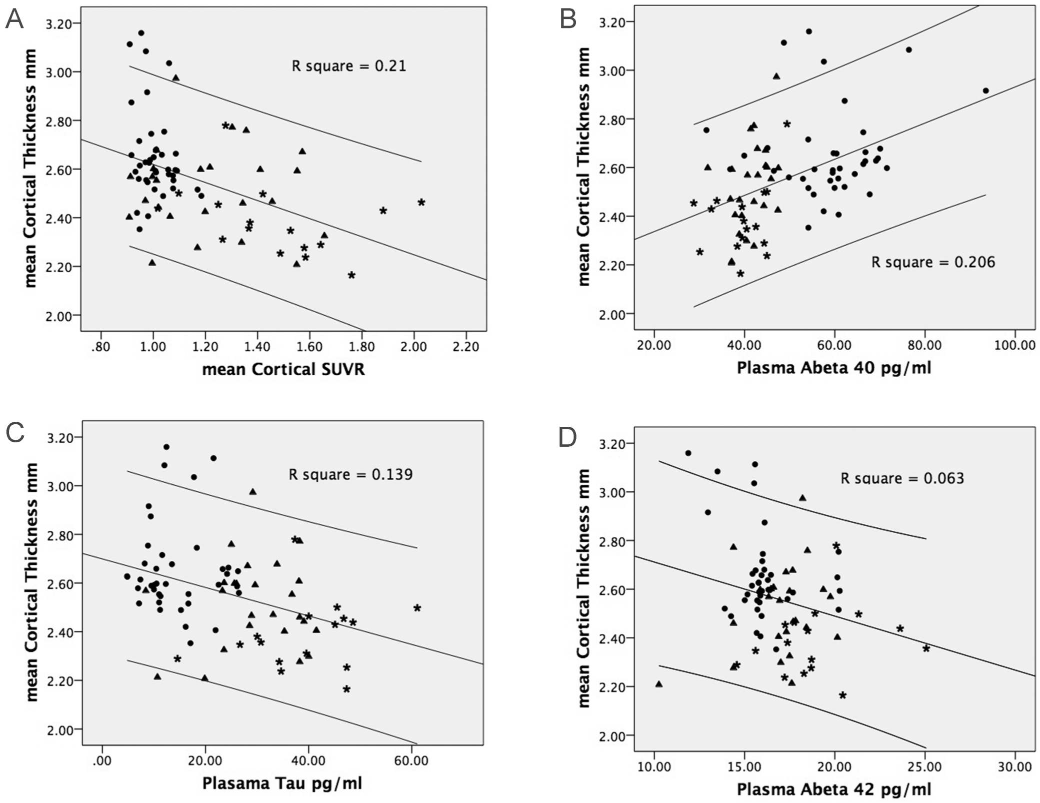

Supplementary Figure 1 A shows univariate linear regression between the mean cortical SUVR and mean cortical thickness (mm); B shows univariate linear regression between plasma $A\beta_{40}$ (pg/ml) and mean cortical thickness (mm); C shows univariate linear regression between plasma tau (pg/ml) and mean cortical thickness (mm); D shows univariate linear regression between plasma $A\beta_{42}$ (pg/ml) and mean cortical thickness (mm); the upper and lower limiting lines define 95% confidence intervals; stars represent patients with Alzheimer's disease, triangles represent subjects with amnesic mild cognitive impairment, and circles represent control subjects.
